# Direct Effects of D-Chiro-Inositol on Insulin Signaling and Glucagon Secretion of Pancreatic Alpha Cells

**DOI:** 10.3390/biom10101404

**Published:** 2020-10-04

**Authors:** Agnese Filippello, Alessandra Scamporrino, Stefania Di Mauro, Roberta Malaguarnera, Antonino Di Pino, Roberto Scicali, Francesco Purrello, Salvatore Piro

**Affiliations:** 1 Department of Clinical and Experimental Medicine, Internal Medicine, Garibaldi-Nesima Hospital, University of Catania, 95122 Catania, Italy; agnese.filippello@gmail.com (A.F.); alessandraska@hotmail.com (A.S.); 8stefaniadimauro6@gmail.com (S.D.M.); antonino.dipino@unict.it (A.D.P.); robertoscicali@gmail.com (R.S.); salvatore.piro@unict.it (S.P.); 2 School of Human and Social Sciences, “Kore” University of Enna, 94100 Enna, Italy; roberta.malaguarnera@unikore.it

**Keywords:** α-cells, D-chiro-inositol, lipotoxicity, insulin resistance

## Abstract

The insulin resistance state of pancreatic α-cells seems to be related to glucagon hypersecretion in type 2 diabetes. Treatment that can improve the insulin sensitivity of α-cells could control glucagon levels in patients with diabetes mellitus. The aim of this study was to investigate the preventive role of D-chiro-inositol (DCI), which has insulin receptor-sensitizer effects on insulin signaling pathways and glucagon secretion in pancreatic α-TC1 clone 6 cells. Cells were chronically treated with palmitate to induce insulin resistance in the presence/absence of DCI. DCI treatment improved the insulin signaling pathway and restored insulin-mediated glucagon suppression in α-TC1-6 cells exposed to palmitate. These results indicate that DCI treatment prevents the insulin resistance of α-TC1-6 cells chronically exposed to palmitate. Our data provide evidence that DCI could be useful to improve the insulin sensitivity of pancreatic α-cells in diabetes treatment.

## 1. Introduction

During the last few years, new interesting knowledge of diabetes mellitus pathophysiology has resulted in important novel therapeutic and clinical implications. More specifically, the clinical use of glucagon-like peptide 1 (GLP-1) analogues or receptor agonists led to pivotal progress in several fields of medicine [1,2]. This cultural progress clarified the roles of gastrointestinal hormones as well as pro-glucagon and α-cells in diabetes pathophysiology [3]. Indeed, it is well known that hyperglycemia in diabetic patients is partly the result of dysregulated high levels of fasting glucagon. In type 2 diabetes (T2D), in contrast to the physiologic state, glucagon hypersecretion arises in hyperglycemic conditions, while glucagon hyposecretion occurs in the hypoglycemic state [4,5], leading to increased plasma glucose and/or prolonged hypoglycemia. These phenomena seem to be related to pancreatic α-cell dysfunction and can be partially explained by insulin resistance in pancreatic α-cells [6].

The altered function and the existence of an insulin resistance state in pancreatic α-cells have been suggested and partially demonstrated in several studies [7,8,9]. On the basis of this evidence, in the past few years, our group has shown that palmitate, which induces lipotoxicity, impairs insulin signaling and glucagon secretion in pancreatic α-cells [10].

Although glucagon dysregulation is as important as insulin dysregulation in type 2 diabetes, therapeutic pharmacological approaches targeting glucagon are lacking [11,12], compared with pharmacological approaches that improve insulin secretion and β-cell function [13,14]. Furthermore, it is still unclear if specific treatments capable of improving the insulin sensitivity of pancreatic α-cells can also control glucagon levels in patients with diabetes mellitus. To date, and to the best of our knowledge, there are no studies concerning the use of drugs or natural therapeutic strategies to prevent and control the insulin resistance state in pancreatic α-cells.

It is widely reported in the literature that several drugs, compounds, molecules, and plant extracts are able to improve the insulin resistance state in humans [15,16]. Among them, D-chiro-inositol (DCI) is commonly used in clinical practice for polycystic ovary syndrome (PCOS), a specific clinical condition in which a profound insulin resistance state underlies the physiopathological mechanisms of the syndrome [17].

DCI is a form of the nine isomers of inositol produced by many plants that have insulin-like properties, acting as secondary messengers in insulin signal transduction [18,19,20]. DCI is often used as a dietary health supplement in the clinical treatment of PCOS and T2D in humans [20,21,22,23,24,25]. Recent studies have indicated that DCI improved glucose metabolism in diabetic mice [26] and increased cellular glucose uptake in skeletal muscle, inducing GLUT4 translocation [27,28]. Finally, it has been demonstrated that DCI induced insulin secretion in β-cells and potentiated glucose-stimulated insulin secretion in mouse islets [29].

To date, it is not clear whether DCI could have a direct effect on pancreatic α-cells. The molecular mechanisms regulated by DCI in α-cells remain to be elucidated.

Since glucagon secretion is regulated by insulin signaling and potentially influenced by an insulin resistance state in pancreatic α-cells, we hypothesized that DCI could influence/prevent insulin signaling pathway regulation in these cell populations. Therefore, the aim of this preclinical study was to investigate the preventive role of DCI in insulin signaling pathways and glucagon secretion in an established model of α-cell insulin resistance.

In order to address our hypothesis, we used an in vitro model of pancreatic α-cells. These cells, as is widely known in the literature, develop an insulin-resistant state after palmitate chronic exposure [10]. In this study, therefore, we investigated whether DCI could prevent palmitate-induced insulin resistance and prevent glucagon secretory dysfunction. Despite this being a preclinical study, our data could contribute to improving the knowledge concerning the role of pancreatic α-cells in diabetes pathophysiology.

## 2. Materials and Methods

### 2.1. Cell Culture

A murine glucagonoma α-TC1 (clone 6) cell line was purchased from the American Type Culture Collection (ATCC; through LGC Standards S.r.l., Milan, Italy). This line was derived from an adenoma created in transgenic mice expressing the SV40 large T antigen oncogene under the control of the rat preproglucagon promoter. α-TC1-6 cells were grown in Dulbecco’s modified Eagle’s medium (DMEM) with 4 mmol/L-glutamine (Sigma-Aldrich, Saint Louis, MO, USA) modified to contain 16.7 mmol/L glucose, supplemented with 10% heat-inactivated dialyzed fetal bovine serum (FBS) (Gibco, Thermo Fisher Scientific, Rodano, MI, Italy), 15 mmol/L HEPES, 0.1 mmol/L nonessential amino acids, and 0.02% bovine serum albumin (BSA) (Sigma-Aldrich, Saint Louis, MO, USA). The cells were passaged once a week and were maintained at 37 °C in a humidified incubator gassed with 5% CO_2_.

### 2.2. Palmitate and DCI Preparation

Palmitate solution (Sigma-Aldrich, Saint Louis, MO, USA) was prepared as previously reported [30] and was diluted in culture medium. DCI was kindly provided by Amicogen Inc. (Jinju, Korea) and shipped by Vivatis Pharma GmbH (Hamburg, Germany). DCI was dissolved in phosphate buffered saline (PBS) (Sigma-Aldrich, Saint Louis, MO, USA) to obtain a concentration of 1 M. The stock solution was filter sterilized and stored at –80 °C, and the DCI solution was freshly diluted in culture medium before each experiment.

### 2.3. MTT and Crystal Violet Assays

Before starting the experiment, in order to mimic lipotoxic metabolic perturbation in the absence of cell death, α-TC1-6 cells were treated with increasing concentrations of palmitate (0.25, 0.5, 0.75, and 1 mM) for 48 h and cell viability was evaluated using 3-(4,5-dimethylthiazol-2-yl)-2,5-diphenyltetrazolium bromide (MTT) and crystal violet (CV) assays. We chose 48 h for palmitate treatment because we previously demonstrated that insulin resistance of α-TC1-6 cells can be induced in this time [10].

MTT assay measures the conversion of MTT to insoluble formazan by dehydrogenase enzymatic activity occurring in the mitochondria of intact living cells [31]. The amount of the product is proportional to the number of living cells and depends on mitochondrial activity. Therefore, MTT is a direct method to determine both cell viability and cytotoxicity and is widely used. Factors influencing formazan production include the cell cycle phase, the phase of cell growth, and some culture conditions such as pH and D-glucose concentration, which may affect cell metabolism [32,33,34].

CV staining does not have limitations that influence the accuracy of MTT or other enzymatic tests. It is a quick and simple nonenzymatic assay that quantifies the total DNA mass of adherent cells and thus determines cell viability directly. The assay is based on the affinity between the dye and the external surface of the DNA double helix. The amount of dye absorbed depends on the total DNA content in the culture, thereby permitting a direct measurement of the number of living cells [34].

An equal number of cells was seeded in 96-well plates and, 24 h after seeding, cells were treated with palmitate. Palmitate supplemented medium was changed every 24 h. Concerning the MTT assay, after incubating with palmitate, 11 µL of MTT solution (5 mg/mL in PBS) was added to the 100 µL culture medium of each well. The plates were further incubated at 37 °C for 4 h, then 100 µL dimethyl sulfoxide (DMSO) was added to each well. Regarding the CV assay, at the end of palmitate treatment, media was aspirated and replaced by a CV stain and then incubated at room temperature for 10 min. The stain was washed off with demineralized water and plates were left to dry [35]. The dye was solubilized by using a mixture of PBS + 1% SDS and DMSO at a 1:1 ratio. Optical absorbance was determined at 570 nm with a microplate spectrophotometer (Victor X3 Multilabel Plate Reader, PerkinElmer, Waltham, MA, USA) for both MTT and CV assays.

MTT and CV assays were also performed to evaluate whether increasing concentrations of DCI (0.1, 0.5, 1, and 2 mM) had a potential cytotoxic effect.

### 2.4. α-TC1-6 Treatment with DCI

Twenty-four hours after planting, α-TC1-6 cells were cultured for 48 h at 37 °C in complete DMEM medium in the presence or absence of both 0.1/1 mM DCI and 0.5 mM palmitate. To assess the effect of insulin, cells were serum-starved for 24 h in medium with 0.1% BSA instead of FBS before being stimulated with insulin. Stimulation with 10^−9^ M insulin (Sigma-Aldrich, St. Louis, MO, USA) was performed for 5 min or 2 h for insulin signaling studies or glucagon secretion analysis, respectively, as previously described [10].

### 2.5. Glucagon Secretion

α-TC1-6 cells were grown in 6-well plates in the presence or absence of DCI (0.1 or 1 mM) and cultured as described above. At the end of this period, glucagon was measured in the supernatants. Cells were then washed and incubated for 2 h in Krebs–Ringer buffer (KRB) containing 16.7 mmol/L glucose and 0.5% BSA (pH 7.4) in the presence or absence of insulin 10^−9^ M. Afterwards, media were collected in tubes containing aprotinin (0.1 mg/L) (Sigma-Aldrich, Saint Louis, MO, USA) and kept frozen at –20 °C for subsequent analysis. The cells were lysed in a radio-immunoprecipitation assay (RIPA) buffer and the lysates were analyzed for total protein content to control for the number of cells, as previously reported [36]. Glucagon levels were measured using a specific ELISA kit (Mercodia Glucagon, Uppsala, Sweden) according to the manufacturer’s instructions.

### 2.6. Cell Lysis, Immunoprecipitation, and Western Blot Analysis

At the end of the culture period, cells were lysed in ice-cold modified RIPA buffer. For insulin receptor (IR) phosphorylation analysis, immunoprecipitation was performed as previously described [37]. Protein A-Sepharose (GE Healthcare Life Sciences, Uppsala, Sweden) was incubated with 2–4 μg of the IR β-subunit (IR-β) antibody (cat. no. Sc-09, mouse monoclonal antibody) (Santa Cruz Biotechnology, Dallas, TX, USA) at 4 °C under constant rotation for 2 h and then overnight with cell lysates. Immunoprecipitates were subjected to SDS-PAGE and then analyzed by immunoblotting with an anti-phospho-insulin receptor β (Tyr1150/1151) antibody (cat. no. 3024, rabbit monoclonal antibody) (Cell Signaling Technology, Danvers, MA, USA).

For Western blot analysis, cell lysates were analyzed as previously described [38]. Specific proteins were detected with the following antibodies: anti-phospho-IRS-1 (Tyr612) (cat. no. 44-816G, rabbit polyclonal antibody) (Thermo Fisher Scientific, Rodano, MI, Italy); anti-total IRS-1 (cat. no. 2382, rabbit polyclonal antibody), anti-phospho-AKT (Ser473) (cat. no. 9271, rabbit polyclonal antibody), anti-total AKT (cat. no. 9272, rabbit polyclonal antibody) (Cell Signaling Technology, Danvers, MA, USA), and anti-β-actin (cat. no. A5441, mouse monoclonal antibody) (Sigma-Aldrich, Saint Louis, MO, USA).

Immunoblot signals were visualized using an Odyssey Fc infrared scanner (LI-COR Biosciences, Lincoln, NE, USA). Densitometric analysis was performed using Odyssey Image Studio Lite v. 5.2 software (LI-COR Biosciences, Lincoln, NE, USA).

### 2.7. Statistical Analysis

All data are presented as means ± SEM. The differences between the means of unpaired samples were analyzed using Student’s t-test. Comparisons between multiple means were performed via an ANOVA followed by post hoc analysis for significance (Bonferroni test). A *p*-value less than 0.05 was considered statistically significant. The statistical analysis was performed using GraphPad Prism 6.0 (GraphPad Software, Inc., San Diego, CA, USA).

## 3. Results

### 3.1. Cell Viability of α-TC1-6 Cells Treated with DCI

**Figure 1:**
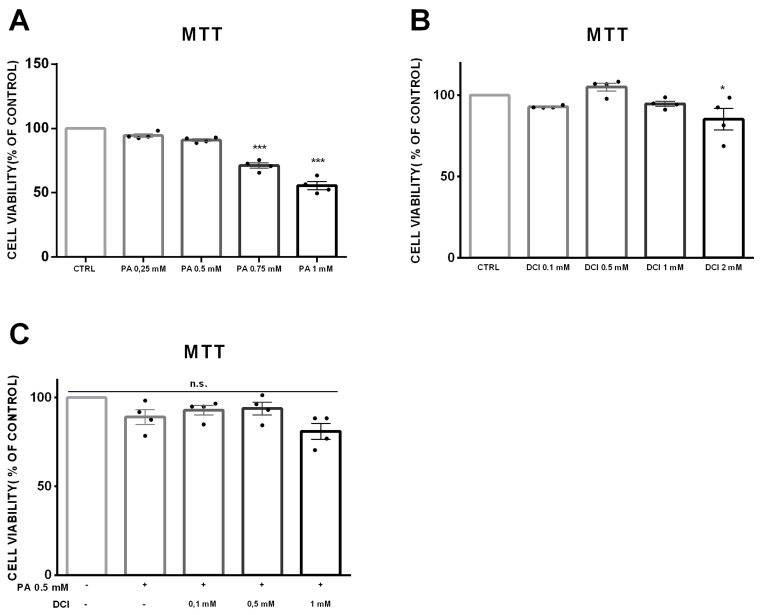
Effect of palmitate and D-chiro-inositol (DCI) exposure on cell viability in α-TC1-6 cells. MTT assay in α-TC1-6 cells exposed to increasing concentrations of palmitate or DCI. (**A**) α-TC1-6 cells exposed to palmitate (0.25, 0.5, 0.75, and 1 mM) for 48 h. (**B**) α-TC1-6 cells exposed to DCI (0.1, 0.5, 1, and 2 mM) for 48 h. (**C**) α-TC1-6 cells exposed to DCI (0.1, 0.5, and 1 mM) in the presence of palmitate (0.5 mM) for 48 h. Data are expressed as scatter plots with bar ± standard error of 570 nM absorbance to percentage of control. Experiments were conducted in four biological replicates. One-way ANOVA followed by Bonferroni test (*n* = 4): * *p* < 0.05, *** *p* < 0.001 with respect to controls. n.s., not significant; PA, palmitate; DCI, D-chiro-inositol.

To test if chronic exposure to palmitate affected cell viability, α-TC1-6 cells were treated with increasing concentrations of palmitate (0.25, 0.5, 0.75, and 1 mM) in the absence of DCI for 48 h. After exposure to 0.25 and 0.5 mM of palmitate, α-TC1-6 cells, in both MTT and CV, showed a minimal but not statistically significant effect on cell viability. In contrast, at 0.75 and 1 mM of palmitate, cell viability was markedly reduced in a dose-dependent manner (Figure 1A, Figure S1). Therefore, we chose the concentration of 0.5 mM palmitate for subsequent experiments.

Thereafter, in order to exclude a potential DCI cytotoxic effect, we performed the above-mentioned in vitro cell viability and cytotoxicity assays on α-TC1-6 cells exposed to increasing DCI concentrations (0.1, 0.5, 1, and 2 mM) for 48 h. DCI did not affect cell viability at concentrations of 0.1, 0.5, and 1 mM, but 2 mM was toxic to α-TC1-6 cells in both MTT (Figure 1B) and CV (Figure S1) assays.

We also analyzed the effect of co-treatment with DCI (0.1, 0.5, and 1 mM) and palmitate (0.5 mM for 48 h) on cell viability. DCI did not modify the effect of palmitate on α-TC1-6 cell viability in both MTT (Figure 1C) and CV (Figure S1) assays.

Based on these results, we chose 0.1 and 1 mM concentrations of DCI to analyze its dose-dependent effect on glucagon secretion in pancreatic α-TC1-6 cells in the absence of palmitate.

In order to validate the absence of toxicity induced by DCI in pancreatic cells, we also performed MTT and CV in the INS-1 β-cell line, a line of β-cells that are a predominant cell population present in islet of Langerhans. INS-1 cells were treated for 48 h with DCI at 0.1, 0.5, 1, and 2 mM. In accordance with the α-cell data, we observed that DCI did not affect cell viability up to 1 mM, while at 2 mM we observed a slight decrease in cell viability (Figure S2).

### 3.2. Effects of DCI Treatment on Glucagon Secretion

**Figure 2:**
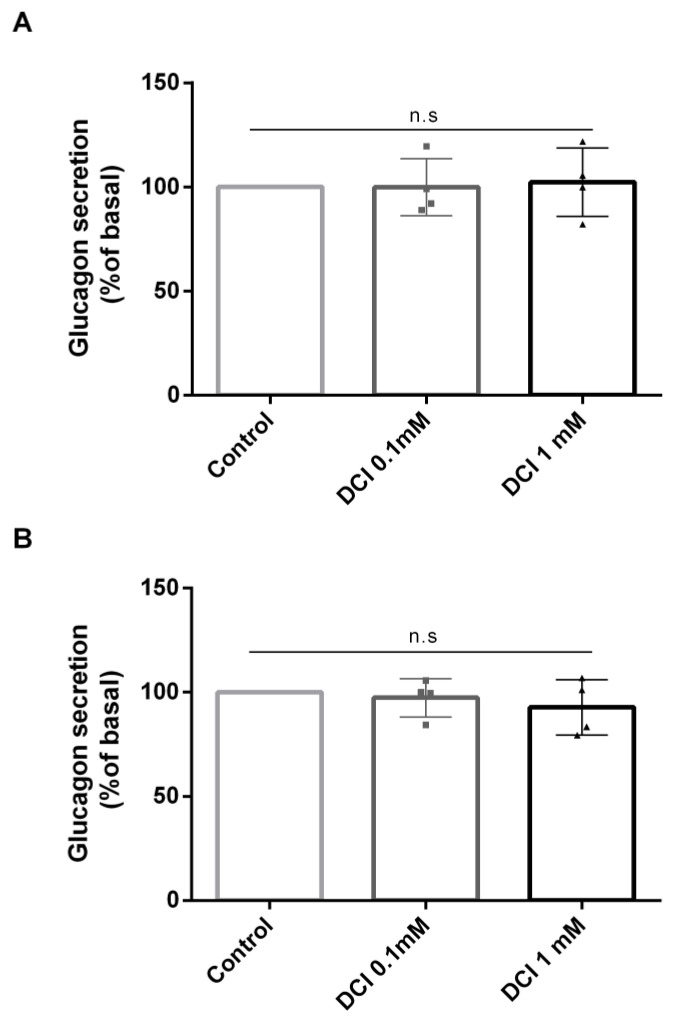
Effect of DCI on glucagon secretion in α-TC1-6 cells. Glucagon secretion in cells treated with DCI (0.1 or 1 mM) for 48 h in control conditions (without palmitate). (**A**) Acute glucagon levels secreted by α-cells cultured in the presence or absence of DCI; glucagon levels were detected after 48 h of treatment in 2 h cell-conditioned Krebs–Ringer buffer (KRB) (control: 1505 pg/mg protein ± 12 vs. DCI 0.1 mM: 1504 pg/mg protein ± 12 vs. DCI 1 mM: 1539 pg/mg protein ± 13). (**B**) Chronic glucagon levels, detected directly in Dulbecco’s modified Eagle’s medium (DMEM) at the end of 48 h of treatment in cells cultured in the presence or absence of DCI in control conditions, without palmitate (control: 1825 pg/mg protein ± 14  vs. DCI 0.1 mM: 1774 pg/mg protein ± 9 vs. DCI 1 mM: 1694 pg/mg protein ± 12). Data represent four independent experiments. One-way ANOVA followed by Bonferroni test (*n* = 4) was used to evaluate statistical significance in DCI treated cells with respect to controls. n.s., not significant; DCI, D-chiro-inositol.

To analyze the effects of DCI on glucagon secretion, α-TC1-6 cells were treated for 48 h with DCI (0.1 and 1mM) in the absence of palmitate. At the end of DCI treatment, cells were washed and cultured for 2 h in KRB using increasing doses of DCI (0.1 and 1mM) and glucagon levels were then measured. As shown in Figure 2, under control conditions (in the absence of palmitate), DCI treatment did not affect the acute glucagon secretion of pancreatic α-TC1-6 cells (Figure 2A).

Furthermore, we measured glucagon levels in the supernatants of α-TC1-6 cells after 48 h of exposure to two doses of DCI (0.1 and 1 mM). DCI treatment slightly reduced the chronic glucagon release of pancreatic α-TC1-6 cells but not in a statistically significant manner (Figure 2B).

In light of these results, we still used 0.1 and 1 mM concentrations to test the dose-dependent effect of DCI on insulin-regulated glucagon secretion and insulin signaling in pancreatic α-TC1-6 cells in the presence of palmitate, mimicking an insulin resistance condition.

### 3.3. Palmitate Induces Insulin Resistance and Impaired Glucagon Secretion

**Figure 3:**
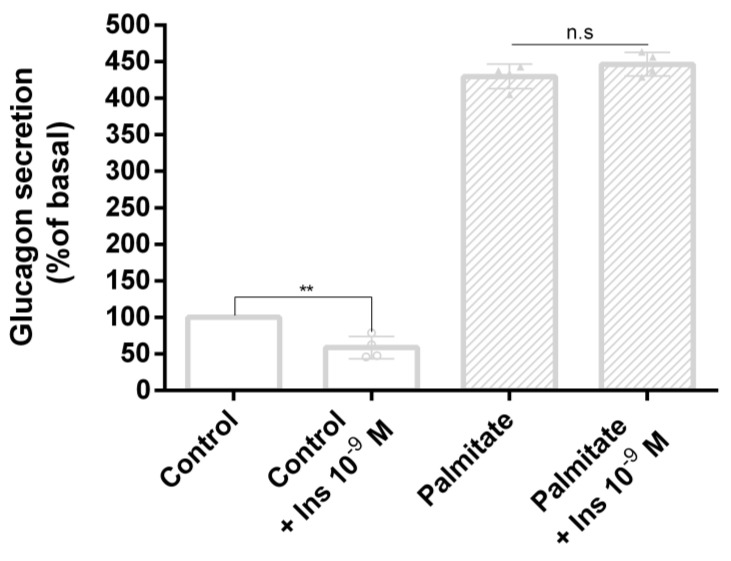
Effect of palmitate on glucagon secretion in α-TC1-6 cells. Insulin-regulated glucagon secretion in control cells and cells treated with palmitate (0.5 mM for 48 h) and/or insulin 10^−9^ M. Data represent four independent experiments. Student’s t-test was used to evaluate statistical significance in insulin-stimulated cells with respect to unstimulated cells in the absence and presence of palmitate (control: 1405 pg/mg protein ± 13  vs. control + Ins 10^–9^ M: 829 pg/mg protein ± 11; palmitate: 6036 pg/mg ± 12  vs. palmitate + Ins 10^–9^ M: 6273 pg/mg protein ± 12 ): ** *p* < 0.01. n.s., not significant.

To evaluate the effect of palmitate on insulin-regulated glucagon secretion, α-TC1-6 cells were treated with palmitate (0.5 mM) for 48 h. Subsequently, after 24 h of serum starvation, cells were washed and cultured for 2 h in KRB in the presence or absence of palmitate (0.5 mM) and/or insulin 10^−9^ M, then glucagon levels were measured. As expected, in control cells, glucagon secretion was inhibited by acute insulin stimulation in a statistically significant manner. In contrast, in cells exposed to palmitate, acute insulin exposure had no effect (Figure 3).

### 3.4. DCI Treatment Prevented Palmitate Effects on Glucagon Secretion

To determine the role of DCI in acute insulin inhibition of glucagon secretion, α-TC1-6 cells were treated with DCI (0.1 and 1 mM) in the presence or absence of palmitate (0.5 mM) for 48 h. After 24 h of serum starvation, cells were washed and cultured for 2 h in KRB in the presence or absence of palmitate (0.5 mM) and/or insulin 10^−9^ M using two doses of DCI (0.1 and 1 mM); at the end of incubation, glucagon levels were measured.

**Figure 4:**
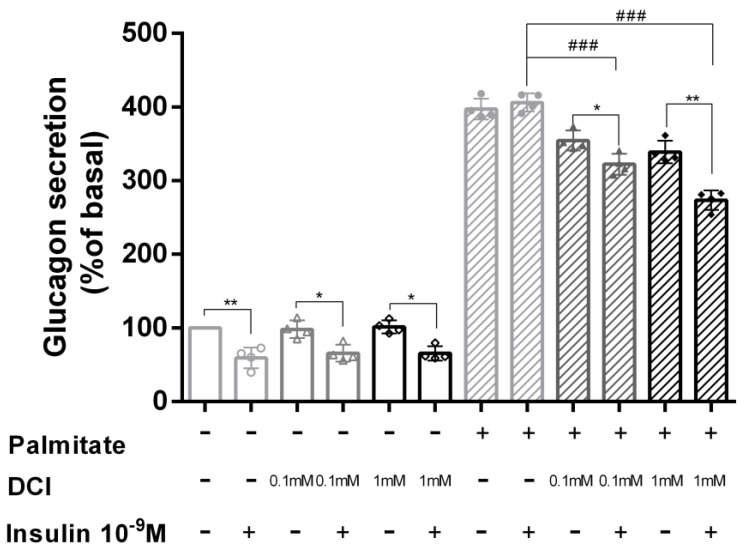
Effect of DCI on glucagon secretion in α-TC1-6 cells exposed to palmitate. Acute insulin inhibition of glucagon secretion in control cells and cells treated with 0.1 or 1 mM DCI in the presence or absence of palmitate (0.5 mM for 48 h) and/or insulin 10^−9^ M. Data represent four independent experiments. Student’s t-test was used to evaluate statistical significance in insulin-stimulated cells with respect to unstimulated cells in the absence and presence of palmitate (control: 1471 pg/mg protein ± 11 vs. control + Ins 10^–9^ M: 870 pg/mg protein ± 10; palmitate: 5852 pg/mg protein ± 10 vs. palmitate + Ins 10^–9^ M: 5976 pg/mg protein ± 9); * *p* < 0.05, ** *p* < 0.01. One-way ANOVA followed by Bonferroni test (*n* = 4) was used to evaluate statistical significance in insulin-stimulated cells exposed to increasing DCI concentrations with respect to insulin-stimulated controls in the presence of palmitate. ^###^ *p* < 0.001. n.s. not significant; DCI, D-chiro-inositol.

As shown in Figure 4, in control cells acute insulin exposure caused a significant inhibition of glucagon secretion in both the absence and presence of 0.1 and 1 mM DCI (control = 41%, *p* < 0.01; 0.1 mM DCI = 34%, *p* < 0.05; 1 mM DCI = 35%, *p* < 0.05). However, in the presence of 0.5 mM palmitate, the inhibitory effect of acute insulin exposure was markedly reduced. In contrast, co-incubation of cells with 0.5 mM palmitate and 0.1 or 1 mM DCI was able to prevent the inhibitory effect of acute insulin exposure on glucagon secretion. More specifically, 0.1 and 1 mM DCI induced a reduction in glucagon secretion of 9% (*p* < 0.05) and 19% (*p* < 0.01), respectively.

### 3.5. Influence of DCI on Insulin Signaling in α-TC1-6 Cells

In order to investigate the molecular mechanisms by which DCI improves the inhibitory action of insulin on glucagon secretion in palmitate-treated α-TC1-6 cells, we analyzed some mediators of the intracellular insulin pathway. We examined the activation of insulin tyrosine kinase receptor (IR) and insulin metabolic pathway components after acute insulin exposure.

**Figure 5:**
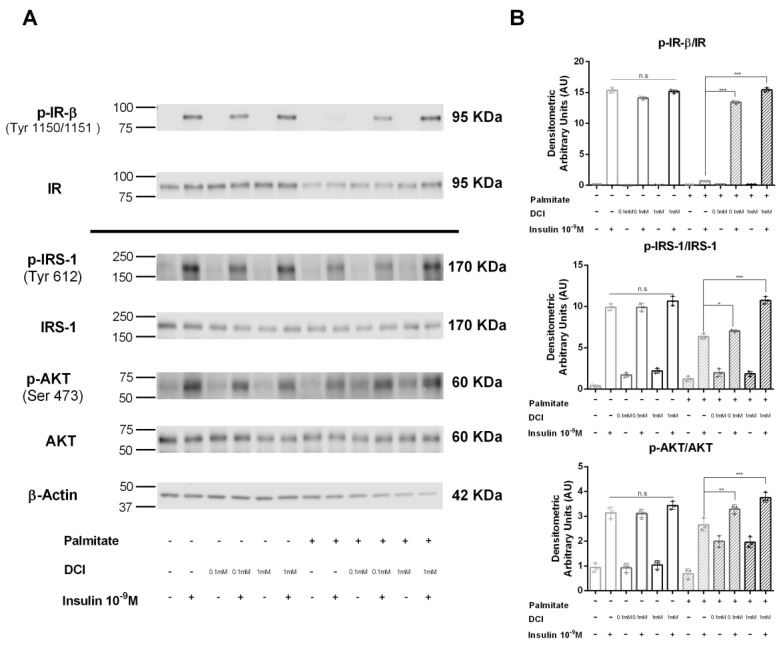
Effect of DCI on insulin receptor (IR) phosphorylation and the IRS-1/AKT pathway in α-TC1-6 cells exposed to palmitate. Immunoprecipitation and Western blot analysis for total IR (Tyr1150/1151-β subunit) (p-IR-β) and total IR; Western blot analysis for p-IRS-1 (Tyr612), total IRS-1 (IRS-1), p-AKT (Ser 473), total AKT (AKT), and β-actin: αTC1-6 cells were treated with DCI (0.1 or 1 mM for 48 h) in the presence or absence of palmitate (0.5 mM for 48 h) and acutely stimulated with insulin (10^−9^ M for 5 min). (**A**) Representative immunoblot of three independent experiments. (**B**) Means ± SEM of densitometric analysis. One-way ANOVA followed by Bonferroni test (*n* = 3) was used to evaluate statistical significance in insulin-stimulated cells treated with increasing concentrations of DCI with respect to insulin-stimulated controls in the absence and presence of palmitate: * *p* < 0.05, ** *p* < 0.01, *** *p* < 0.001. n.s., not significant; DCI, D-chiro-inositol.

As shown in Figure 5, in control cells, acute stimulation with 10^−9^ M insulin for 5 min induced a significant increase in tyrosine phosphorylation of the IR β subunit (p-IR-β), whereas in palmitate pre-exposed cells, the insulin effect on IR phosphorylation was abrogated; moreover, in palmitate pretreated cells, the contemporaneous presence of DCI (0.1 and 1 mM) markedly increased insulin-induced IR-β phosphorylation, with an increasing trend with respect to cells treated only with palmitate in the absence of DCI (Figure 5A).

Subsequently, we studied IRS-1 phosphorylation (Tyr612) (p-IRS-1) by using Western blot analysis. In control cells, insulin significantly stimulated IRS-1 phosphorylation. In contrast, in palmitate pre-exposed cells, this effect was markedly reduced; in this condition, the presence of DCI (0.1 and 1 mM) significantly improved the effect of insulin on IRS-1 phosphorylation (Figure 5A).

Finally, we evaluated AKT phosphorylation (Ser473) (p-AKT) in palmitate pre-exposed cells; the presence of DCI (0.1 and 1 mM) significantly increased insulin-induced AKT phosphorylation compared to cells exposed only to palmitate (Figure 5A).

## 4. Discussion

In this study we analyzed the role of DCI in glucagon secretion. We demonstrated that DCI is able to prevent palmitate-induced effects on intracellular insulin signaling in pancreatic α-cells. In our results, chronic treatment (48 h) with palmitate (0.5 mM) altered intracellular insulin signaling and made α-cells not respond to acute insulin stimulation; DCI was able to prevent such alterations, preventing the onset of insulin resistance and insulin unresponsiveness of α-cells. We used the clonal α-TC1-6 cell line, which has the advantage of being a homogeneous cellular population with respect to primary islets; furthermore, it has been used previously to study glucagon secretion and protein expression [10,39].

By using DCI, we observed that it is possible, in our system, to prevent α-cell secretory dysfunction. Since α-cell secretory dysfunction underlies diabetes mellitus pathophysiology, and although these data are preliminary and the experiment was conducted on a cellular line, they are relevant and important to lay a cultural foundation for future research in this field [12,40]. In order to fully understand these aspects, it is important to note that, in diabetes, α-cells do not respond to secretory or inhibitory stimuli (i.e., insulin or glucose) in a coordinated manner. Therefore, hyperglucagonemia is observed in hyperglycemic conditions, while a reduced counterregulatory response has been reported in hypoglycemic conditions, mainly in insulin-treated patients [41,42]. During the natural history of diabetes, these aspects, in addition to negatively affecting β-cells, also cause functional alterations in α-cells. Functional alterations reported in α-cells seem to have a more important impact with respect to β-cell loss during diabetes. Indeed, deregulated glucagon levels have a pivotal role already at the onset of diabetes and result in a greater effort made by the whole organism to restore glycemic homeostasis [11,43].

The key role of glucagon in diabetes pathogenesis has largely been described by Professor Roger Unger in his scientific production, and α-cell dysfunction and insulin unresponsiveness have been suggested and researched [4,44,45,46,47,48]. Nevertheless, since α-cells are rarely represented in pancreatic islets, direct functional assays are very difficult to perform [42,49,50]. Therefore, the use of in vitro models such as the α-TC1 cell line represents a good approach to overcome this experimental difficulty.

Our research group demonstrated for the first time the key role of insulin signaling in pancreatic α-cells and the deleterious effects of palmitate in a previous study [10]. The results in the present study suggest the use of DCI to prevent palmitate-induced alterations in pancreatic α-cells. Consequently, DCI, used in patients with insulin resistance, could exert a direct beneficial effect on pancreatic α-cells. Our data demonstrate that DCI has a potential prevention effect vs. palmitate. To date, there are several diabetes drugs that indirectly act on glucagon secretion in various ways: the administration of GLP-1 reduces glucagon secretion in patients with T2D [51], and metformin treatment, which improves hepatic insulin resistance, suppresses glucagon secretion [52]; nevertheless, the direct effects of these treatments on glucagon secretion and α-cell function remain to be elucidated. Moreover, the effects of alternative drugs or natural therapeutic strategies on insulin resistance control and prevention need to be investigated. In our study, we showed, for the first time, the direct effect of DCI treatment on insulin-regulated glucagon secretion in α-TC1-6 cells exposed to palmitate; in these cells, the presence of DCI improved insulin-mediated glucagon suppression with an increasing trend.

Currently, many studies have shown that alternative therapies can improve insulin secretion, hyperglycemia, and insulin sensitivity in animal models of T2D, in β-cell lines and in T1D [53,54,55,56,57,58,59]. Regarding pancreatic α-cells, Chen et al. demonstrated that isosteviol treatment reduced the hypersecretion of glucagon and restored α-cell dysfunction induced by palmitate [60]. In this study, we show that another alternative therapy, DCI treatment, can improve α-cell function after chronic exposure to palmitate; we demonstrated that DCI improves the insulin sensitivity of α-TC1-6 cells chronically treated with palmitate.

Several studies reported that DCI can reduce hyperglycemia and improve insulin resistance [24,61,62]; the administration of DCI, for example, reduced hyperglycemia and hypertriglyceridemia in diabetic patients [63]. Nevertheless, the mechanism underlying the antidiabetic effects of DCI remains largely unclear; for this purpose, we studied the influence of DCI treatment on insulin signaling pathways in pancreatic α-cells. In our findings, we showed that DCI treatment was able to restore phosphorylation of the IR/IRS-1/AKT pathway in α-TC1-6 cells exposed to palmitate, ameliorating the insulin responsiveness of α-cells. Previous studies indicated that DCI can improve the insulin pathway by acting on the IR/AKT axis in hepatic and skeletal muscle tissues [27,64,65].

In summary, our data indicate that DCI treatment prevents insulin resistance of α-TC1-6 cells, an in vitro model of α-cells, chronically exposed to palmitate. Specifically, these cells showed an improvement of the IR/IRS-1/AKT insulin signaling pathway in the presence of DCI. Consequently, exposure to DCI restored insulin-mediated glucagon suppression in palmitate-induced insulin-resistant α-TC1-6 cells.

A limitation of our study is that we used an immortalized cell line model and, the same as all studies performed on in vitro cell models, αTC1-6 cells cannot fully mimic α-cells in vivo. Nevertheless, αTC1-6 cells have an advantage over primary islets, as they represent a homogeneous cellular population. Furthermore, whole pancreatic islets contain a very low percentage of α-cells, and consequently their isolation and the subsequent study of glucagon secretion are very difficult. Therefore, in our opinion, this aspect could represent an advantage for our study. Indeed, if we had used whole islets, we could not have isolated the specific effect of DCI on α-cells, and the results would represent a cumulative effect derived from the interaction between all cell types present in the pancreatic islets. Certainly, another limit of our work is that these data require more preclinical and clinical validation. Although DCI is normally used in the clinical treatment of women with PCOS and to treat insulin resistance during pregnancy [17,66], the use of DCI to treat insulin resistance of α-cells in diabetic patients needs appropriate clinical trials.

In spite of the limitations reported above, this study shows that DCI plays a positive role in regulating insulin-mediated glucagon secretion through the IR/IRS-1/AKT signaling pathway. These results help in identifying a specific cellular and molecular mechanism responsible for diabetes pathophysiology.

## 5. Conclusions

These findings shed new light on the direct effect of DCI on the insulin inhibition of glucagon secretion, including the molecular mechanisms associated with the dysfunction of α-cells after chronic exposure to palmitate. These data, although produced in an in vitro model, could support data obtained in diabetic patients; therefore, our data could contribute to the indication of a new specific treatment that can improve the insulin sensitivity of pancreatic α-cells and control glucagon levels in patients with diabetes mellitus.

## Supplementary Materials

The following are available online at https://www.mdpi.com/2218-273X/10/10/1404/s1, Figure S1: Effect of palmitate and DCI exposure on cell viability in α-TC1-6 cells. Figure S2: Effect of DCI exposure on cell viability in INS-1 cells.

## Abbreviations

AKT	Serine-threonine protein kinase
CV	Crystal violet
DCI	D-chiro-inositol
GLP-1	Glucagon-like peptide-1
IR	Insulin receptor
IRS	Insulin receptor substrate
KRB	Krebs–Ringer buffer
MTT	3-(4,5-dimethylthiazol-2-yl)-2,5-diphenyltetrazolium bromide
PCOS	Polycystic ovary syndrome
T2D	Type 2 diabetes
