# Supplementary material for: Direct Effects of D-Chiro-Inositol on Insulin Signaling and Glucagon Secretion of Pancreatic Alpha Cells
Source: Biomolecules. 2020 Oct 4;10(10):1404. doi: 10.3390/biom10101404 (PMC7601246; doi:10.3390/biom10101404)
Supplement: Supplementary file 1 [file biomolecules-10-01404-s001.pdf]

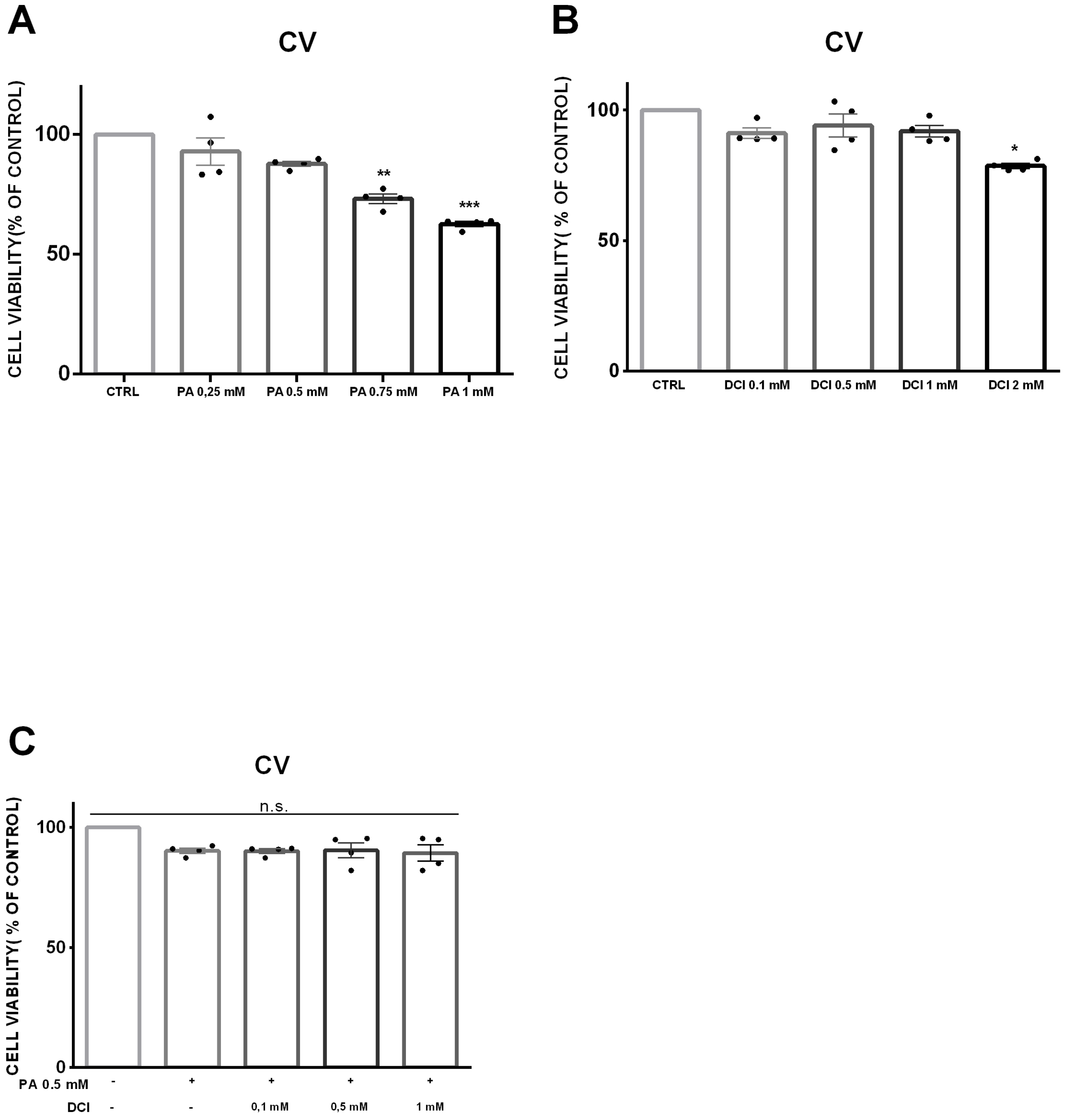

Figure S1. Effect of palmitate and DCI exposure on cell viability in α-TC1-6 cells.

Crystal violet assay in α-TC1-6 cells exposed to increasing concentrations of palmitate or DCI. (**A**) α-TC1-6 cells exposed to palmitate (0.25 mM, 0.5 mM, 0.75 mM and 1 mM) for 48 h. (**B**) α-TC1-6 cells exposed to DCI (0.1 mM, 0.5 mM, 1 mM and 2 mM) for 48 h. (**C**) α-TC1-6 cells exposed to DCI (0.1 mM, 0.5 mM and 1 mM) in the presence of palmitate (0.5 mM) for 48 h. Data are expressed as scatter plots with bar ± standard error of 570 nM absorbance to % of control. Experiments were conducted in four biological replicates. One-way ANOVA test: *$p < 0.05$, ** $p < 0.001$, *** $p < 0.0001$ with respect to controls. n.s., not significant; PA, palmitate; DCI, D-chiro-inositol; CV, Crystal Violet.

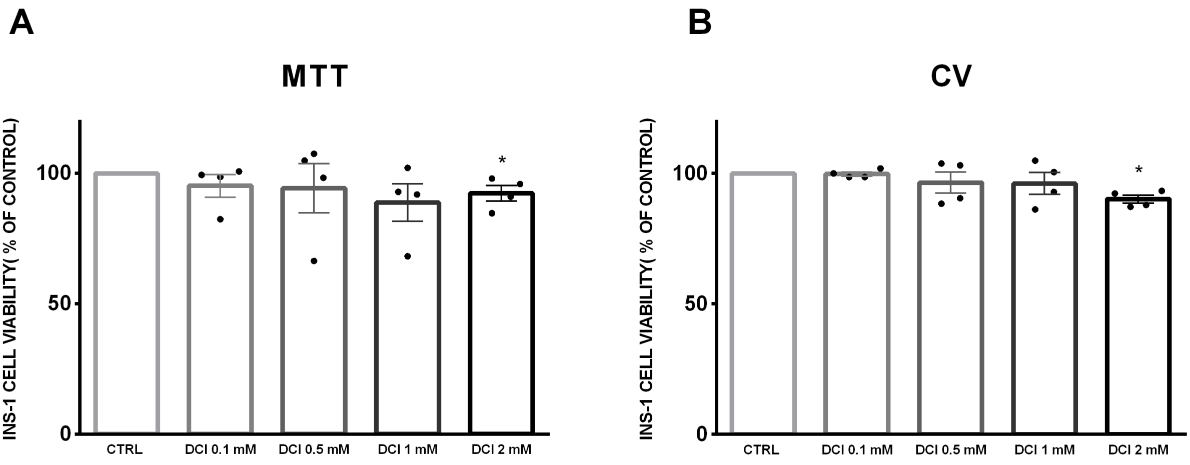

Figure S2. Effect of DCI exposure on cell viability in INS-1 cells.
MTT assay (**A**) and crystal violet assay (**B**) in INS-1 cells exposed to increasing concentrations of DCI (0.1 mM, 0.5 mM, 1 mM and 2 mM) for 48 h. Data are expressed as scatter plots with bar ± standard error of 570 nM absorbance to % of control. Experiments were conducted in four biological replicates. One-way ANOVA test: * p <0.05 with respect to controls. PA, palmitate; DCI, D-chiro-inositol; CV, Crystal Violet.
